# Public health round-up

**DOI:** 10.2471/BLT.17.010717

**Published:** 2017-07-01

**Celebrating Dengue Day in south-east Asia**

People sway to the grooves of the Dengue Dance in the Philippines on the Association of South EastAsian Nations’ (ASEAN) Dengue Day, which is celebrated every year on 15 June in Brunei Darussalam, Cambodia, Indonesia, Lao People’s Democratic Republic, Malaysia, Myanmar, the Philippines, Singapore, Thailand and Viet Nam to raise awareness about dengue prevention and control.

http://asean.org/asean-dengue-day/

**Figure:**
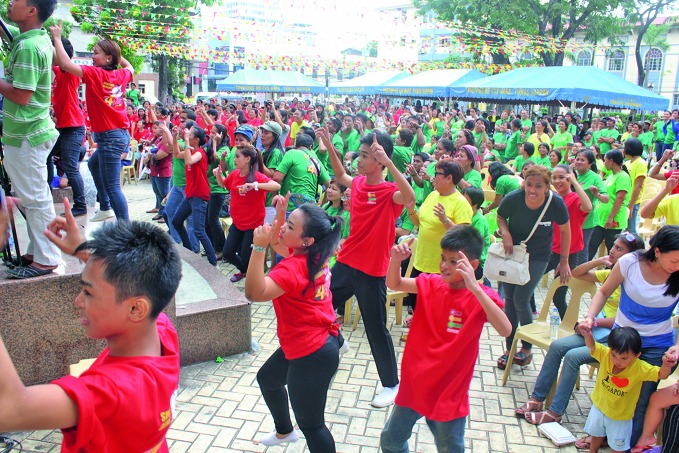
Sanofi Pasteur/Elarcosa Photo and Video Services

## New WHO Director-General takes office

Dr Tedros Adhanom Ghebreyesus took office this month as the new Director-General of WHO following his election by WHO’s Member States at the World Health Assembly in May of this year. He was nominated by the Government of Ethiopia and selected under new procedures introduced in 2016. 

As Ethiopia’s Minister of Health 2005–2012, he led comprehensive reform of the country's health system, including an expansion of the health infrastructure, creating 3500 health centres and 16 000 health posts. He expanded the health workforce by 38 000 health extension workers and initiated financing mechanisms to expand health insurance coverage. 

He has also served as chair of the boards of the Global Fund to Fight AIDS, Tuberculosis and Malaria and the Roll Back Malaria Partnership, as well as being co-chair of the Board of the Partnership for Maternal, Newborn & Child Health.

He succeeds Dr Margaret Chan who has been WHO’s Director-General since 1 January 2007.

http://www.who.int/mediacentre/news/releases/2017/director-general-elect/

## Data on attacks on health 

A total of 302 attacks on health facilities, health workers and ambulances were reported across 20 countries last year, according to data gathered by WHO, in which 372 people died and 491 people were injured. 

Such attacks have serious consequences for health service delivery, often killing and injuring patients and providers, and depriving people of care in conflict-affected countries. 

Most (207/302) of the attacks were reported in the Syrian Arab Republic, followed by 20 in Libya, 18 in the Central African Republic, 11 in the West Bank and Gaza Strip and nine in the Democratic Republic of the Congo. Two thirds or 207 were on health-care facilities, 52 on health workers, 40 on ambulances and three on patients. 

WHO started analysing reports on attacks on health care, in countries facing emergencies, from secondary sources and consolidating these data in 2014. According to a report released last year based on these data, WHO reported 256 such attacks in 2015 and 338 in 2014. The data are not comprehensive but give an idea of the magnitude of the problem. 

 “While we recognize that we are not capturing all data, and that there is significant underreporting, this is the only existing consolidation of global data on individual attacks on health care in emergency contexts,” the statement said.

These attacks on health workers and health care often contravene international humanitarian law. WHO condemned the attacks and continues to advocate for an end to such attacks in all settings.

http://www.who.int/emergencies/attacks-on-health-care

## Cholera resurgence 

WHO and its partners have been responding to a resurgence of cholera in parts of Yemen that has claimed more than 601 lives and caused more than 73 762 cases as of 1 June.

WHO distributed medicines and medical supplies, including cholera kits, oral rehydration solutions and intravenous fluids as well as medical furniture and equipment for diarrhoea treatment centres. 

The centres have been rolled out across 18 of Yemen’s 19 target governorates that are affected by cholera, according to Dr Nevio Zagaria, WHO Representative in Yemen.

The cholera outbreak in Yemen was announced by Yemen’s health ministry in October 2016. An estimated 7.6 million people live in areas at high risk of cholera.

The resurgence comes as Yemen’s already weakened health system struggles under the weight of two years of conflict. Key infrastructure, including water and sanitation facilities, is collapsing, contributing to the spread of diarrhoeal disease. 

The weather is also playing a role: the pathogens that cause cholera are more likely to spread in warmer weather and recent heavy rains have washed piles of uncollected waste into water sources.

“We are very concerned with the re-emergence of cholera across several areas of Yemen in the past couple of weeks. Efforts must be scaled-up now to contain the outbreak and avoid a dramatic increase in cases of diarrhoeal disease,” Zagaria said. 

Cholera is an acute diarrhoeal infection caused by ingestion of food or water contaminated with the bacterium *Vibrio cholera* that can be fatal. 

http://www.emro.who.int/media/news/who-responds-to-resurgent-cholera-in-yemen.html

**Cover photo**

To address climate change and other issues affecting the world’s oceans seas, a United Nations conference was held in New York from 5 to 9 June to support the implementation of sustainable development goal 14: conserve and sustainably use the oceans, seas and marine resources for sustainable development. The photo shows fishermen in Viet Nam.

**Figure:**
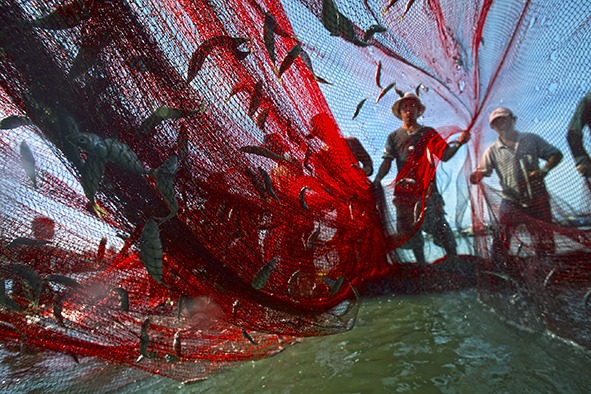
© Tran Vinh Nghia/UNDP

## Human rights and health 

A joint WHO and Office of the United Nations High Commissioner for Human Rights (OHCHR) report released at the 70th World Health Assembly in May, urged political leaders to anchor the provision of health care for women, children and adolescents in their countries more firmly in human rights principles.

The report, entitled *Leading the realization of human rights to heath and through health*, was prepared by the High-Level Working Group on the Health and Human Rights of Women, Children and Adolescents established a year ago. It provides guidance on how countries should implement human rights-related measures called for under the* Global strategy on women’s, children’s and adolescents’ health (2016−2030)*. 

The report makes nine recommendations to governments to create a transformative leadership agenda to improve the health and well-being of women, children and adolescents. 

One recommendation urges countries to uphold the right to health – enshrined in the WHO constitution – in national law, another calls on countries to allocate at least 5% of gross domestic product for public health spending, which is needed for universal health coverage.

The recommendations also urge governments to prohibit and adequately punish gender-based violence, end female genital mutilation and child, early and forced marriage, and to remove barriers to the realization of sexual and reproductive health and rights. 

The Global Strategy, launched by the United Nations Secretary-General and global leaders, alongside the sustainable development goals in September 2015, is a roadmap for ending the preventable deaths of women, children and adolescents, and ensuring that they can reach their full potential and transform their own futures and those of their communities.

http://www.who.int/life-course/publications/hhr-of-women-children-adolescents-report

## Priority antimicrobials 

The 5th revision of WHO’s list of critically important antimicrobials published in May 2017 ranks antibiotics according to their importance in human medicine. 

The list enables stakeholders in the agriculture sector and regulatory agencies to prioritize efforts to ensure the prudent management of these critical medicines in agriculture. This will help to better control the rise of antibiotic resistant bacteria in those sectors. 

The updated version entitled *Critically important antimicrobials for human medicine 5th Revision 2016* divides antimicrobial classes into three priority groups of critically important, highly important and important. 

The list was first published in 2005 in response to increasing calls for action to curb antimicrobial resistance associated with the food chain. 

Antibiotics are grouped in classes and categorized as “critically important” when this class represents one of very few therapies or the only therapy capable of treating certain serious bacterial infections in people, and are used to treat infections where the bacteria or their resistance genes may be transmitted to humans from nonhuman sources. 

“Critically important” antibiotics include those that are frequently used in humans and where a large number of people are affected by bacterial diseases for which the antibiotic is the sole or one of few alternatives to treat the infections in humans. Currently, the highest priority critically important antimicrobials are cephalosporins (3rd and higher generation), glycopeptides, macrolides and ketolides, polymyxins and quinolones

http://www.who.int/foodsafety/publications/antimicrobials-fifth

## Cancer devices list 

WHO has released a list of basic and priority medical devices required for the management of cancer as a reference for low- and middle-income countries that are facing a growing disease burden. 

The *WHO list of priority medical devices for cancer management* includes medical devices for the prevention, screening, diagnosis, treatment and palliative care related to six types of cancer: breast, cervical, colorectal, leukaemia, lung and prostate.

These devices range from surgical instruments, laboratory equipment and personal protective equipment to quality assurance devices and radiation protection devices. 

http://www.who.int/medical_devices/publications/priority_med_dev_cancer_management/en/

**Looking ahead**

1−3 November − World Hepatitis Summit 2017, São Paulo, Brazil

18−20 October − WHO Global Conference on Noncommunicable Diseases, Montevideo, Uruguay

16−17 November − Global Ministerial Conference on Tuberculosis, Moscow, Russian Federation

